# Maternal Cardiac Function During Pregnancy in Predicting Preeclampsia: A Narrative Review

**DOI:** 10.3390/biomedicines14102211

**Published:** 2026-09-29

**Authors:** Anwar H. Nassar, Elie Hobeika, Shorouk Hajeer, Ihab Usta

**Affiliations:** Department of Obstetrics and Gynecology, American University of Beirut Medical Center, Beirut 1107, Lebanoniu00@aub.edu.lb (I.U.)

**Keywords:** preeclampsia, onset, cardiac function, parameters, predictors, narrative review

## Abstract

**Background/Objectives**: Preeclampsia is a major hypertensive disorder that affects pregnant women. It is a leading cause of maternal morbidity and mortality. The exact underlying pathophysiological mechanisms that lead to the occurrence of preeclampsia are still uncertain; however, many studies have underscored several maternal contributing factors. Our review focuses on maternal cardiac hemodynamic parameters in predicting the onset of preeclampsia. **Results**: Multiple studies have scrutinized the association between preeclampsia and maternal factors including cardiac function. Numerous maternal cardiac hemodynamic parameters were highlighted as significant predictors of the onset of preeclampsia including cardiac output, stroke volume, mean arterial pressure, systematic vascular resistance, and uterine artery pulsatility index. These parameters, except for cardiac output, were significantly increased in women with preeclampsia in comparison to normotensive ones, with mild discrepancies in literature findings concerning SVR. Moreover, twin pregnancies were more likely to develop preeclampsia, and clear discrepancies were reported in maternal cardiac function parameters based on parity status and early- versus late-onset preeclampsia. **Conclusions**: Our findings suggest that maternal cardiac hemodynamics could be crucial predictors of preeclampsia. However, due to the heterogeneity evident across all included studies that limited our synthesis of the findings, further research is needed to reach more valid and confirmatory results in order to incorporate the necessary clinical measures that would aid in the prevention and timely management of preeclampsia.

## 1. Introduction

Preeclampsia is a multisystemic syndrome that complicates 2–4% of pregnancies worldwide [1,2]. It is clinically defined as new-onset hypertension (≥140/90 mmHg) in a previously normotensive woman and proteinuria (≥0.30 g/24 h) after 20 weeks of gestation [3]. Preeclampsia continues to be a major leading cause of maternal morbidity and mortality, with an estimated maternal mortality rate of 16% that is attributed to preeclampsia and other related hypertensive disorders of pregnancy [4]. While the case fatality remains relatively low (<1%) in high-resource settings, it can be as high as 10–25% in severe untreated cases, specifically in low-resource settings [5]. The underlying pathophysiology is fairly complicated. One of the proposed physiological mechanisms assumes that impaired placentation, which results from the improper remodeling of spiral arteries, leads to an elevated resistance in placental blood flow. Subsequently, chronic placental ischemia manifests, with a significantly decreased blood flow to the fetus [6].

Several major maternal and neonatal health complications have been associated with preeclampsia. Eclampsia, HELLP syndrome (hemolysis, elevated liver enzyme levels, and low platelet count), abruption placentae, intracerebral hemorrhage, and acute kidney injury are some of the short-term maternal complications encountered in women diagnosed with preeclampsia [7]. Long-term complications include chronic hypertension, chronic renal insufficiency, diabetes mellitus, and mental illnesses [6]. At the fetal–neonatal level, short-term complications involve higher rates of intrauterine growth restriction (IUGR), intrauterine fetal death (IUFD), oligohydramnios, preterm birth and its complications, low birth weight, cerebral palsy (CP), and neonatal intensive care unit (NICU) admission [7]. In the long run, children of women with preeclampsia encounter more neuropsychological and behavioral disorders, learning difficulties, chronic respiratory illnesses, recurrent infections, and metabolic diseases [7].

Given the severity of preeclampsia and its health consequences, investigating the risk factors that drive its onset is an urgent research priority. Multiple risk factors have been discussed in the literature, with varying risk ratios (RR) including antiphospholipid syndrome (RR: 9.72), history of preeclampsia (RR: 7.19), pre-gestational diabetes (RR: 3.56), multigravidity (RR: 2.93), nulliparity (RR: 2.91), family history of preeclampsia (RR: 2.9), and extremes of age whether adolescence or age ≥ 40 years (RR: 1.96) [8]. Other risk factors include inherited thrombophilia, pre-pregnancy body mass index (BMI) > 30 kg/m^2^, kidney disease, medical disorders including connective tissue diseases and hyperthyroidism, and assisted reproduction [9].

Cardiovascular disease has been highlighted as another plausible risk factor for developing preeclampsia. A case–control observational study revealed that pregnant women with valvular heart disease prior to pregnancy have an increased risk of developing preterm preeclampsia [10]. Several studies have assessed the cardiac function of pregnant women who eventually developed preeclampsia during the different trimesters of pregnancy. An observational study explored cardiac function in 4795 pregnant women midgestational, 126 of whom subsequently developed preeclampsia. Multivariate analysis showed that the vascular resistance was higher, while the left ventricular global longitudinal systolic strain, cardiac output (CO), and ejection fraction were lower among women who developed preeclampsia in comparison to those who did not [11]. Moreover, across a total of 2764 women with singleton pregnancies who were assessed for their cardiac function at 35–37 weeks of gestation, those who developed preeclampsia were reported to have a significantly higher vascular resistance and mean arterial pressure (MAP) associated with a decreased cardiac output [12]. In another study, out of 1602 women with singleton pregnancies, 50 (3.12%) developed preeclampsia. Women with a third trimester echocardiographic index E/e′ ≥ 7.3 and an increased left ventricular mass indexed for a body surface area ≥ 63.2 g/m^2^ were more likely to develop preeclampsia than their counterparts [13].

As evident in the literature, there is a possible association between maternal cardiac function during pregnancy and preeclampsia. Most of the current evidence probes maternal cardiovascular health after the onset of preeclampsia, examining the implications of preeclampsia on maternal cardiac outcomes. Therefore, the primary objective of this narrative review is to gather the evidence regarding the clinical ability of surveilling maternal cardiac function during gestation in predicting the onset of preeclampsia.

## 2. Methods

A non-systematic search of the literature was conducted using PubMed, ScienceDirect, Ovid Embase, Cochrane Library, and Google Scholar to navigate studies and gather eligible ones. “Preeclampsia”, “maternal cardiac function”, and “cardiac hemodynamics” were the primary search terms utilized. There was no time limit on the selected studies, but all of the articles included were published between the years 2000 and 2026. Studies were deemed eligible if they investigated the association between maternal cardiac function and preeclampsia, regardless of the study design and exact parameters scrutinized.

## 3. Parameters of Maternal Cardiac Function, During Gestation, in Association with the Onset of Preeclampsia

Multiple maternal cardiac parameters have been studied as predictive variables for the onset of preeclampsia. It is quite important to emphasize the heterogeneity across all studies and their results based on multiple variables, including gestational age, which will be discussed.

### 3.1. Cardiac Output

Cardiac output (CO) refers to the total volume of blood pumped through the heart per minute [14]. It is a byproduct of stroke volume (SV) and heart rate [14]. Women undergo fundamental cardiovascular changes during pregnancy that are reversed in the few weeks postpartum [15]. An increase in blood volume is significantly detected, starting by the sixth week of gestation, reaching a peak range of 4700–5200 mL (45% total increase in blood volume is typically reported) [15]. A meta-analysis gathered data from 39 studies that assessed maternal CO in healthy women with a singleton pregnancy. It concluded that the CO reached a peak value of 6.48 L/min, which reflects a 1.5 L/min increase in comparison to the value reported for non-pregnant women [16]. Similarly, another systematic review estimated an increase in the CO that started from the early weeks into gestation, and the maximal mean difference was registered during the third trimester of gestation (1.41 L/min) [17].

In light of the above, the CO is anticipated to be significantly altered in women who develop hypertensive disorders of pregnancy, namely preeclampsia. There is however a discrepancy between studies in the literature, as some have reported a decreased CO as a predictor to developing preeclampsia, while others reported the opposite. Numerous observational studies concluded that women who eventually developed preeclampsia had decreased CO in comparison to those who did not [11,12,18,19,20,21] (Table 1). A study assessed Log10 CO for women at low and high risk for developing preeclampsia throughout their clinical visits during gestation. A significant steady decline was estimated for women at a high risk for developing preeclampsia; however, women at a low risk had an increase until the third visit, followed by a decrease [22]. On the other hand, a few observational studies reported an increased CO among the group of women who developed preeclampsia [23,24] (Table 1). Conversely, other studies did not report an association between the CO and preeclampsia (*p*-value > 0.05) [25,26].

### 3.2. Stroke Volume (SV)

SV represents the volume of blood expelled out of the left ventricle per one systolic contraction [27]. It is usually measured by the difference between blood volume in the ventricle at the end of a diastole (end diastolic volume) and that at the end of a systole (end systolic volume) [28]. During pregnancy, profound changes affect the cardiovascular functions of women, which include the SV that gradually increases throughout gestation [29]. Studies have marked a 30% total increase in SV, compared to non-pregnant levels [30]. However, with the predisposition of hypertensive disorders of pregnancy, like preeclampsia, these changes are exacerbated. Compared to pregnant women who remain normotensive, the SV is noticeably increased among women who develop preeclampsia [18,23,24,31] (Table 1). In a study that examined maternal cardiac function during passive leg raising, a significantly higher increase in SV (7.27 mL) was noted in hypertensive women compared to normotensive women (2.14 mL); *p*-value < 0.01 [21]. Other studies, on the other hand, showed that the increase in SV was not statistically significant (*p*-value > 0.05) [13,19,22,32].

**Table 1:** Literature findings regarding cardiac output and stroke volume.

Reference	Sample Size (Without Preeclampsia/with Preeclampsia)	Cardiac Output (L/Min)		Cardiac Output (Multiple of the Median)			Stroke Volume (mL/Beat)			Gestational Age (Weeks)
		Without Preeclampsia	With Preeclampsia	Without Preeclampsia	With Preeclampsia	*p*-Value	Without Preeclampsia	With Preeclampsia	*p*-Value	
Gibbone et al. 2022 [11]	4557 */126			1.0 (0.99–1.006) #	0.96 (0.93–0.99) #	0.02				19–23
Guy et al. 2017 [12]	2586 */66	6.8 ± 1.01	6.3 ± 0.9			<0.025				35–37
Tiralongo et al. 2015 [18]	75/25	7.17 ± 0.4	5.5 ± 0.9			<0.01	70.04 ± 12.43	91.23 ± 12	<0.01	5–11
Pisani et al. 2017 [19]	223/5	7.9 ± 1.3	6.7 ± 0.47			<0.05				6–36
Gagliardi et al. 2017 [20]	138/12	6.7 ± 1.1	5.6 ± 0.3			<0.001				11–13
Liang et al. 2019 [21]	314/16	6.28 ± 1.38	5.71 ± 1.18			<0.001				22–24
Khaw et al. 2008 [23]	457/8	4.9 (4.3–5.5)	6.2 (5.4–7.1)			<0.01	67.0 ± 11.7	87.9 ± 15.2	<0.001	11–14
Mansukhani et al. 2024 [24]	6746/176	6.88 (6.84–6.91)	7.27 (7.06–7.49)			<0.05	77.2 (76.9–77.6)	85.3 (82.3–88.5)	<0.05	35–37
Valensise et al. 2008 [31]	1345/107						83 ± 11	102 ± 19	<0.05	20+

* Total number of unaffected patients; excluding 112 patients with gestational hypertension. # Left ventricular cardiac output.

### 3.3. Mean Arterial Pressure

Mean arterial pressure (MAP) refers to the average arterial pressure per one cardiac cycle; a systole and a diastole. It is affected by two main variables: CO and systematic vascular resistance (SVR) [33]. MAP undergoes substantial alterations during gestation. It decreases with pregnancy, with the greatest decrease occurring in the early weeks of gestation (sixth–eighth week) [34]. However, evidence demonstrated that women at higher risk of developing hypertensive disorders have a higher MAP than low-risk women [11,13,21,23,24,32,35,36,37] (Table 2). Moreover, a study measured Log10 MAP to estimate the change in MAP throughout a total of four different clinic visits during pregnancy (first visit: 11–14; second visit: 19–24; third visit: 30–34; fourth visit: 35–37 weeks’ gestation). In women who developed preeclampsia, Log10 MAP was significantly higher than that for normotensive women [22].

### 3.4. Systematic Vascular Resistance

SVR is the force applied by the vasculature of the body on the circulating blood [38]. To be more explicit, SVR is the quantitative value for the afterload of the left ventricle [39]. This force is impacted by three major factors: the length of the blood vessels, diameter of the vessels, and viscosity of blood [38]. Over the course of a normal pregnancy, the SVR decreases considerably, especially at its early stages, and it continues to decrease in the second and third trimesters [40]. A meta-analysis concluded that the SVR continuously decreased to its nadir in the early stages of the third trimester, but it increased modestly at term [17].

A few studies have documented lowered levels of SVR among women who developed preeclampsia compared to normotensive women [18,23,31], while others showed elevated levels of SVR [11,13,19,20,21,24,32] (Table 2). Yet, one study that measured Log10 vascular resistance did not document a significant difference between both groups of women [22].

### 3.5. Uterine Artery Pulsatility Index

Uterine artery pulsatility index (UAPI) measures the impedance to blood flow across the arteries which supply the fetus. It is derived from the uterine artery waveform on doppler ultrasound (peak systolic velocity − minimum diastolic velocity)/time-averaged velocity) [41]. This index helps in detecting disorders encompassing hypertensive disorders, associated with placental dysfunction [41]. UAPI is often high at early pregnancy, followed by a progressive decrease [42]. A prospective longitudinal cohort study of normotensive women with singleton pregnancies investigated the normal ranges of UAPI based on gestational age. It was concluded that the UAPI was 1.84 ± 0.55, 1.07 ± 0.38, and 0.78 ± 0.23 across the first, second, and third trimester, respectively [42]. Conversely, the onset of hypertensive disorders is prone to change the UAPI.

Varying studies have simultaneously reported a higher UAPI in comparison to normotensive women 1.10 (0.88–1.34) vs. 0.98 (0.8–1.20) (19–23 weeks of gestation) [11], 1.12 ± 0.55 vs. 0.84 ± 0.25 (22–24 weeks of gestation) [21], 1.14 ± 0.47 vs. 0.68 ± 0.13 (24–40 weeks of gestation) (*p*-value < 0.001) [43]. In contrast, a prospective cross-sectional study documented a higher UAPI among women with preeclampsia (1.72 ± 0.5 vs. 1.87 ± 0.5; 11–14 weeks of gestation), but these values did not reach statistical significance [23]. Furthermore, UAPI was a significant predictor for the onset of preeclampsia [24], with an increased risk reported for women who had an elevated UAPI measured at the 12th week of gestation (*p*-value = 0.026) [44]. In addition, the odds ratio of developing preeclampsia was 5.99 (1.64–21.13) and 10.32 (2.75–42.49), among women with an abnormally elevated UAPI (>95th percentile) in the first and third trimester of gestation [45].

### 3.6. Left Ventricle Metrics

#### 3.6.1. Left Ventricular Mass Index

The left ventricular mass is measured via echocardiography. It represents the weight of the left ventricle, and it is related to body size [46]. Normal pregnancy is characterized by an increase in the left ventricular mass. A cross-sectional study aimed to estimate the change in left ventricular mass during normotensive pregnancy and reported a 52% increase in the left ventricular mass [47]. However, in women with hypertensive disorders of pregnancy, this increase is more striking. A prospective observational study deduced a significant difference in left ventricular mass between normotensive pregnant women and those who developed preeclampsia, with the following respective values: 61.2 (54.1 to 68.5) and 64.6 (58.6 to 71.4) (*p*-value = 0.001) [13]. A retrospective cohort study also revealed that a higher left ventricular mass of >61 g/m^2^ is correlated with the later onset of preeclampsia [37].

#### 3.6.2. Left Ventricular Ejection Fraction

The left ventricular ejection fraction (LVEF) is a critical measurement of the left ventricle’s systolic function. It estimates the percentage of blood that leaves the left ventricle per heartbeat [48]. A prospective cross-sectional study revealed that the LVEF was lower among pregnant women at a late stage compared to early stage of pregnancy or non-pregnant women, but the reported values were within a normal range [49]. In contrast, a prospective observational study aimed to predict the onset of preeclampsia by relying on cardiovascular indices at 19–23 weeks of gestation, and the median value of LVEF was found to be significantly lower among women who developed preeclampsia than normotensive women (*p*-value = 0.046) [50]. However, other studies did not identify any significant difference [51].

#### 3.6.3. Left Ventricular Systole and Diastole

Both the systolic and diastolic functions of the left ventricle are subject to alterations during pregnancy. A study that examined left ventricular hemodynamics in normal pregnancy showed that women suffered from a diastolic dysfunction portrayed by decreased early diastolic flow velocity, decreased E wave/A wave ratio, and increased isovolumetric relaxation and deceleration time [52]. However, all these parameters restored their normal levels by 8 weeks postpartum. Similarly, a longitudinal study investigated left ventricular systolic and diastolic long-axis function in normal pregnancy utilizing doppler tissue imaging. This study emphasized decreased early diastolic velocity, but the systolic velocity and E/E0 ratio were not significantly changed [53]. A major diastolic dysfunction was reported among women who developed preeclampsia in a study that compared maternal cardiovascular function in normotensive and hypertensive pregnancies [32]. While using the American Society of Echocardiography 2009 criteria, almost half of the women with preeclampsia were associated with grade II or grade III diastolic dysfunction, while the systolic function was quite similar in both groups [32]. Additionally, in a prospective study, left ventricular global longitudinal systolic strain was significantly lower across women who had preeclampsia (*p*-value = 0.026) compared to those who had an uneventful pregnancy, reflecting a lower cardiac systolic function, although end-diastolic dimensions were comparable in both study groups [22].

### 3.7. Other Parameters

Besides the above findings, some studies have underlined a few other cardiovascular parameters that are associated with the onset of preeclampsia. In a retrospective cohort study, developing preeclampsia was significantly correlated with higher interventricular septal thickness (>7.87 mm; *p*-value = 0.003) and left atrial anteroposterior diameter (>33.5 mm; *p*-value < 0.001) versus normotensive pregnancies [37]. Another study measured a lower atrial area in pregnant women with preeclampsia compared to normotensive ones. With multivariate regression modeling, this significantly contributed to the onset of preeclampsia (*p*-value = 0.016) [22].

### 3.8. Singleton Versus Twin Gestation

It is clear that maternal cardiac function is significantly impacted in singleton gestation. The reported changes in maternal cardiovascular hemodynamics are expected to be even further aggravated in twin gestation. A prospective study probed maternal cardiac function across uncomplicated twin pregnancies, compared with singleton pregnancies. In twin gestations, a significantly higher CO (6.55 ± 0.82 vs. 5.62 ± 0.82 L/min at 20–23 weeks; *p*-value = 0.007) and a much lower SVR (1005 ± 137 vs. 1179 ± 199 dynes/sec/cm^5^ at 20–23 weeks; *p*-value = 0.009) were reported [54]. A study failed to show significant differences in several cardiac parameters in twin gestation with preeclampsia and those without (*p*-value > 0.05) [55]. Conversely, a cross-sectional study found that twin gestation with preeclampsia had a significantly increased SV and CO, higher left ventricular mass, and lower E/A ratio compared to twin gestation without preeclampsia and singleton pregnancies with preeclampsia [56].

### 3.9. Parity (Nulliparas Versus Multiparas)

Parity plays a critical part in dictating the variations in maternal cardiac parameters. Multiparas were found to have a significantly higher CO (5.6 versus 5.2 L/min; *p*-value < 0.001) and median cardiac index (2.3 versus 2.1 L/min/m^2^; *p*-value < 0.001) versus nulliparas. These changes are a reflection of a higher SV (73.5 versus 70.5 mL; *p*-value < 0.001), heart rate (76 versus 75 bpm; *p*-value = 0.003), and left ventricular outflow diameter (20.4 versus 20.0 mm; *p*-value < 0.001), in addition to a lower SVR (1190.8 versus 1253.7 dynes/sec/cm^5^; *p*-value < 0.001) and UAPI (1.6 versus 1.7; *p*-value = 0.003) [57]. In hypertensive pregnancies, multiparas had higher log-mean CO compared to nulliparas (*p*-value = 0.001) [58].

### 3.10. Early Versus Late Preeclampsia

The timing of preeclampsia onset has its own implication on the trajectory of maternal cardiovascular hemodynamics. Early-onset preeclampsia (prior to 34 weeks of gestation) is associated with maternal and perinatal adverse outcomes, whereas late-onset preeclampsia is correlated with more favorable outcomes [59]. In a study by Valensise et al., women with early-onset preeclampsia were found to have higher SVR (1605 ± 248 versus 739 ± 244 dynes/sec/cm^5^) and lower CO (4.49 ± 1.09 versus 8.96 ± 1.83 L) (*p*-value < 0.001) than women with late-onset preeclampsia [31]. Another study reported the difference in mean arterial blood pressure between the 20th and 37th week of gestation to be significantly higher for women with late-onset preeclampsia than those with early-onset preeclampsia (13.3 versus 1.2; *p*-value = 0.031) [36].

## 4. Discussion

Pregnant women are subject to monumental physiological changes, which affect cardiovascular functions. Hypertensive disorders of pregnancy magnify the alterations in cardiovascular hemodynamics, which subsequently contribute to adverse maternal and neonatal outcomes. Therefore, investigating predictors of these disorders at an early stage is critical to effective prevention and timely management. Our narrative review primarily focused on probing the underlying predictors of preeclampsia, as depicted by cardiac parameters. CO, SV, MAP, SVR, and UAPI were concluded as probable predictive parameters to the onset of preeclampsia. All these parameters showed significant elevation in women with preeclampsia compared to normotensive women except for a decrease in CO in the preeclampsia group.

### 4.1. Comparison with the Literature

Various reviews have attempted to explore multiple obstetric and maternal parameters that could predict the onset of preeclampsia. A review gathered 68 prediction models from a total of 70 studies that included 425,125 participants and reported that the most tested predictors in those models encompassed blood pressure (51%) and UAPI (49%) [60]. Another systematic review and meta-analysis focused on MAP and blood pressure metrics in predicting the onset of preeclampsia. The areas under the receiver operator characteristic curves for women at a low risk of developing preeclampsia were 0.68 for systolic pressure, 0.66 for diastolic pressure, and 0.76 for MAP. Among high-risk women, a diastolic pressure of ≥75 mm Hg in the 13th–20th week of gestation was the primary factor that predicted the onset of preeclampsia, with a positive and negative likelihood ratio of 2.8 and 0.39, respectively [61]. Furthermore, a systematic review and meta-analysis underscored the clinical ability of UAPI in predicting preeclampsia. Of the 27 total studies included in the review, the pooled sensitivity and specificity of UAPI were 0.586 and 0.879, respectively, indicating a moderate sensitivity and high specificity [62].

### 4.2. Interpretation of Findings

Mounting evidence suggests that women who develop preeclampsia experience a decrease in CO compared to normotensive women. This is attributed to the vasoconstrictive nature of this disorder that is similar to typical state of hypertension. In normal pregnancies, an initial increase in CO and a decrease in SVR protects against hypertension [63]. However, as this autoregulation falls short in preventing hypertension, CO decreases and SVR increases [64]. This hypothesis is referred to as the cross-over postulate, as the hemodynamics of CO and SVR transition from one state to the opposite. This explanation is based on data from pioneering studies by Easterling et al. [64] and Bosio et al. [65].

The initial rise in CO early in pregnancy in women who will develop hypertensive disorders is probably related to a significant increase in heart rate, rather than in SV, indicating sympathetic overactivity to neutralize hemodynamics changes during the first trimester [17]. Subsequently, this overexertion in sympathetic activity results in an increase in myocardial contractility, thus leading to an increase in SV [66].

The increase in SVR noted in preeclampsia could be attributed to the release of placental factors triggered by reduced uteroplacental perfusion pressure and subsequent placental ischemia. These factors launch a cascade of cellular and molecular processes that result in dysfunctional endothelial and smooth muscle cell, thus increasing vascular resistance and arterial pressure [67]. Another probable explanation for the increase in arterial pressure that is so far only validated in animal models could be related to the significant renal changes encountered in preeclampsia [67].

The proposed interpretation for the relationship between increased resistance to the flow of the uterine artery (increased UAPI) and developing preeclampsia is related to the compromised trophoblast invasion that causes placental impairment [68,69]. High values of UAPI reflect an elevated resistance in uterine artery, which indicates that the remodeling of maternal spiral arteries, responsible for sustaining normal placental blood flow, is insufficient [70]. This hindered remodeling ability of spiral arteries could lead to uteroplacental ischemia, thus triggering the onset of preeclampsia [71,72,73].

The elevated ventricular mass index found in pregnant women with preeclampsia, compared to normotensive women, indicates that the heart is adapting to the increased afterload caused by heightened blood pressure, which is typically observed in preeclampsia [74]. The significant increase in SVR aggravates remodeling of the left ventricle, leading to a moderate dysfunction of left ventricular diastole and concentric hypertrophy [75]. This type of hypertrophy, unlike the eccentric one, suggests an abnormal maternal cardiac adaptation to the increase in SVR [75].

As delineated, there are explicit discrepancies between early- and late-onset preeclampsia. These differences can be attributed to placental vascular remodeling that occurs at early-onset preeclampsia, which is often associated with elevated SVR and decreased levels of SV and CO, whereas late-onset preeclampsia might be correlated with maternal metabolic syndrome, leading to a more benign appearing preeclampsia [18].

Women with a twin gestation are 2–3 times more likely to develop preeclampsia than women with a singleton pregnancy, and they are often characterized by an early onset and faster progression [76]. The disparity in maternal cardiac parameters between singleton and twin gestations is primarily attributed to the additive strain of twin gestation on maternal cardiovascular health [56]. As for parity, nulliparas are at almost twice the risk to develop preeclampsia than multiparas [77]. This can be attributed to the previous immune adaption to novel paternal antigens, which were activated in previous pregnancies, and are more rapidly triggered in a subsequent pregnancy [57].

## 5. Conclusions

To the best of our knowledge, this is the first narrative review that scrutinized maternal cardiac function for its potential role in predicting the onset of preeclampsia including major cardiac parameters such as the cardiac output, stroke volume, mean arterial pressure, systematic vascular resistance, and uterine arterial pulsatility index. Since our review is narrative, our conclusions could have been biased, especially since the findings were based on a qualitative appraisal of the literature. It highlights current gaps in the literature and calls for further research on the topic focusing on maternal cardiovascular hemodynamics as a plausible predictor of preeclampsia while integrating several cardiac parameters. Future studies need to homogenize their probed parameters and methods to enable a quantitative pooling of findings, in addition to carrying out statistical modeling to assess the impact of those variables in developing preeclampsia. Once validated, those results can be built on to incorporate the necessary clinical measures to aid in the prevention and early management of preeclampsia through close monitoring and the possible manipulation of different maternal cardiac parameters.

## Figures and Tables

**Table 2:** Literature findings regarding mean arterial pressure and systematic vascular resistance.

Reference	Sample Size (Without Preeclampsia/with Preeclampsia)	Mean Arterial Pressure (mmHg)			Systematic Vascular Resistance (Dynes/Sec/cm^5^)		Systematic Vascular Resistance (Multiple of the Median)			Gestational Age (Weeks)
		Without Preeclampsia	With Preeclampsia	*p*-Value	Without Preeclampsia	With Preeclampsia	Without Preeclampsia	With Preeclampsia	*p*-Value	
Gibbone et al. 2022 [11]	4557 */126	84.4 (79.7–89.5)	92.6 (87.0–97.3)	<0.0001	1221 (1214–1229)	1329 (1279–1381)			<0.0001	19–23
Garcia-Gonzalez et al. 2020 [13]	1602/50	87.9 ± 6.82	97.6 ± 5.53	<0.001	1400 (1202–1630)	1500 (1393–1831)			<0.001	35–37
Tiralongo et al. 2015 [18]	75/25				1362.2 ± 173.7	1029.7 ± 88.8			<0.01	5–11
Pisani et al. 2017 [19]	223/5				1150 ± 229	1341.6 ± 131.1			<0.05	6–36
Gagliardi et al. 2017 [20]	138/12				1061.4 ± 179.5	1279.8 ± 166.4			<0.001	11–13
Liang et al. 2019 [21]	314/16	78.23 ± 6.99	88.67 ± 14.31	<0.0001	984.24 ± 212.26	1243.94 ± 394.18			<0.001	22–24
Khaw et al. 2008 [23]	457/8	77.3 (72.7–83.3)	90.0 (82.0–91.0)	<0.0001	1260 (1110–1460)	1105 (920–1280)			<0.01	11–14
Mansukhani et al. 2024 [24]	6746/176	85.9 (81.2–91.1)	97.9 (93.5–104.0)	<0.0001			1.00 (0.997–1.007)	1.11 (1.08–1.13)	<0.05	35–37
Valensise et al. 2008 [31]	1345/107				990 ± 179	739 ± 244			<0.05	20+
Pillai et al. 2022 [32]	40/40	85.7 (83.4–87.9)	112.1 (107.8–116.4)	<0.001	2277.2 (1888.8–2665.6)	2867.6 (2517.1–3218.0)			0.035	20+
Mayrink et al. 2019 [36]	1165/87	84.9 (82.3–87.6)	94.2 (92.4–96.0)	<0.01						20+
Xiang et al. 2023 [37]	54/105	97 ± 7	100 ± 6	0.001						20+

* Total number of unaffected patients; excluding 112 patients with gestational hypertension.

## Data Availability

No new data were created or analyzed in this study. Data sharing is not applicable to this article.
